# Role of Calcium Propionate and Monensin on Performance, Rumen Fermentation Patterns, and Ruminal Bacterial Populations in Growing Lambs

**DOI:** 10.3390/vetsci12040298

**Published:** 2025-03-24

**Authors:** Amada Isabel Osorio-Terán, German D. Mendoza, Luis A. Miranda-Romero, Daniel Martínez-Gomez, Pedro A. Hernández-García, Velia Verónica Rangel-Ramírez, Héctor A. Lee-Rangel

**Affiliations:** 1 Departamento de Producción Agrícola y Animal, Universidad Autónoma Metropolitana—Xochimilco, Mexico City CP 04960, Mexico; aosorio@unpa.edu.mx (A.I.O.-T.); gmendoza@correo.xoc.uam.mx (G.D.M.); dmartinez@correo.xoc.uam.mx (D.M.-G.); 2 Universidad del Papaloapan, Campus Loma Bonita, Oaxaca CP 68400, Mexico; 3 Departamento de Zootecnia, Universidad Autónoma Chapingo, Texcoco. Km. 14.5, Texcoco CP 56230, Mexico; albertomiranda@correo.chapingo.mx; 4 Centro Universitario Amecameca, Universidad Autónoma del Estado de México, Amecameca CP 56900, Mexico; pahernandezg@uaemex.mx; 5 Facultad de Agronomía y Veterinaria, Centro de Biociencias, Universidad Autónoma de San Luis Potosí, San Luis Potosí CP 78321, Mexico; veronica.rangel@uaslp.mx

**Keywords:** ionophore, methane, rumen fermentation, rumen bacteria

## Abstract

The objective of this study was to determine the effects of dietary calcium propionate (CaPr) and monensin (MON) on lamb growth performance, ruminal volatile fatty acids (VFAs), methane (CH_4_) production, and ruminal bacteria and archaea populations. Forty crossbred ram lambs (initial weight 23.4 ± 2.8 kg) were randomly assigned to one of four diets (n = 10 per treatment): (a) control diet (CONT); (b) MON diet, which contained 30 mg/kg dry matter (DM) of monensin + CONT; (c) CaPr diet, which contained 10 g/kg DM of CaPr + CONT; and (d) MCA diet, which contained both additives (30 mg/kg DM of monensin plus 10 g/kg DM of CaPr) + CONT. Dietary MON or CaPr in lamb diets did not affect (*p* ≥ 0.05) dry matter intake (DMI), average daily gain (ADG), or DMI/ADG. The main effect (*p* ≥ 0.05) of CaPr supplementation was observed in acetate concentration and CH_4_ production. The addition of calcium propionate increased (*p* < 0.05) *Fibrobacter succinogenes*, *Ruminococcus albus*, and *Selenomonas ruminantium*, whereas CaPr reduced (*p* < 0.05) methanogenic archaea. The addition of MON and CaPr did not affect lamb performance; however, CaPr improved fermentation kinetics towards more efficient routes, reflected by a reduction in the proportion of methane and methanogenic archaea.

## Simple Summary

Animal production is looking for new alternatives to use as additives, ingredients, or supplements to increase productivity. These alternatives should also have a sustainable focus. This study explores the effect of calcium propionate (CaPr) and monensin (MON) and their potential to be used as supplements in ruminants. It seeks modifications to the rumen microbiome that can affect its species-specific modulations without affecting its performance. Lamb performance was not affected by CaPr or MON. However, the fermentation patterns showed that ruminal acetate production decreased with CaPr supplementation, reducing methane ruminal production. Interestingly, CaPr increased some important bacterial species such as *Fibrobacter succinogenes*, *Ruminococcus albus*, and *Selenomonas ruminantium*. Still, the interaction with MON decreased the metanogenic archeas population and total ruminal bacteria.

## 1. Introduction

Livestock will face enormous challenges in mitigating and adapting to climate change in the coming years, as the growing population’s demand for meat and milk is expected to increase [1]. Intensive livestock farming is a significant source of greenhouse gas emissions to the atmosphere, enteric fermentation being one of the primary sources of methane produced [2]. Methane is a potent greenhouse gas, with a global warming potential 34-times stronger than CO_2_ [3]. Enteric methane is a natural byproduct from microbial fermentation of nutrients in the digestive tracts of ruminants [4]. Enteric methane emissions represent up to 11% of the gross energy of the diet consumed by ruminants [5]. The emission of enteric CH_4_ depends on several factors. Efforts to decrease CH_4_ emissions by ruminants focus on diet manipulation, either by increasing the proportion of starch in the diet, including lipids, or using feed additives [2,6,7]. Ionophores are additives traditionally used to improve ruminant productive performance with a concurrent reduction in enteric CH_4_ emission [8]. Ionophores primarily inhibit Gram-positive bacteria and some acetate-producer species [9] that change VFA profiles, although they can inhibit some Gram-negative bacteria in the rumen [10].

Consequently, ionophores could reduce methane emissions by decreasing H_2_ production [11]. Appuhamy et al. [12] reported that a 10% reduction in methane emission was observed using monensin (MON) in dairy and beef diets. Lee-Rangel et al. [13] reported that adding 10 g/kg DM maintains the same gain and feed efficiency associated with an increased rumen propionate. However, Miranda-Romero et al. [14] reported that using calcium propionate in ruminant diets does not affect the in vitro ruminal propionate concentration from the methane produced [14]. The effects of additives on methane are associated with changes in rumen microorganisms that can be evaluated through molecular techniques that provide an opportunity to quantify rumen microbes with high sensitivity and precision [15] and confirm the effects on methanogenic archaea.

Methanogenic archaea are strictly anaerobic microorganisms found in the digestive tract of ruminants and termites, river and lake sediments, swamps, and rice fields. Methanogens colonize the rumen rapidly even before the diet contains forage material. Populations reach their maximum density (109 cells/mL) around day 21 of life [16]. Rumen methanogenic archaea are strictly anaerobic organisms requiring oxygen-free conditions and a reduced oxide potential lower than −330 mV. Some rumen archaea need coenzyme M involved in the final step of methanogenesis to transform the methyl group (CH3) to CH_4_. However, others can synthesize it, such as *Methanobrevibacter* spp. [17]. Methanogenic bacteria constitute a special class in the rumen population because they regulate total fermentation by removing H_2_. The H_2_ removal by methanogenic species stimulates important H_2_-producing species such as *Ruminococcus albus*, *Ruminococcus flavefasciens*, and *Selenomonas ruminantium* to produce more H_2_, thus altering their metabolism towards pathways with higher energy yields [18]. The CH_4_ production by these microorganisms is part of their energy metabolism. Most use CO_2_ as their terminal electron acceptor in anaerobic respiration, converting it to CH_4_; the electron donor used in this process is generally H_2_.

We hypothesize that adding ionophores to the ration will reduce enteric methane emissions and improve the productivity of finishing lambs. However, when combined with calcium propionate the effect will be potentialized and will favor the reduction in methane, thus improving dietary energy utilization. Therefore, the objective of this study was to determine the effects of dietary calcium propionate and monensin on lamb performance, ruminal fermentation kinetics, and ruminal bacteria (*Fibrobacter succinogenes, Ruminococcus albus*, *Genus Prevotella*, and *Selenomonas ruminantium*) and archaea (methanogenic archaea) populations. To achieve this study’s objective, *Fibrobacter succinogenes* and *Ruminococus albus* were chosen as they are related to cellulolytic activities. *Prevotella* was chosen as one of the most abundant rumen bacterial genera. At the same time, *Selenomonas ruminantium* is a fumarate reducer and denitrifier that has a crucial role in H_2_ sinks in sheep. Lastly, methanogenic archaea were chosen due to their role in methanogenesis.

## 2. Materials and Methods

### 2.1. Ethics

The animals were cared for and handled according to the recommendations of the Animal Care and Use Committee of the Universidad Autónoma del Estado de México (Project ID AIOT-0211-2017).

### 2.2. Animals, Feeding, and Management

This study was conducted at the Centro Universitario Amecameca of Universidad Autónoma del Estado de México, Research Experimental Station, Mexico. Forty crossbred male lambs (Suffolk x Pelibuey) (initial weight 23.4 ± 2.8 kg) were randomly assigned to one of four experimental diets (n = 10 per treatment): (a) control diet (CONT); (b) MON diet, which contained 30 mg/kg dry matter (DM) of monensin (Rumensin^®,^ Elanco, Indiana, United Satates) + CONT; (c) CaPr diet, which contained 10 g/kg DM of CaPr (Alimentaria Mexicana Bekarem, Mexico) + CONT; and (d) MCA diet, which contained both additives (30 mg/kg DM of monensin plus 10 g/kg DM of CaPr) + CONT (Table 1). Diet was offered as a total mixed ration. These doses were selected based on the results of previous studies by our research group [13,14]. The rations were modified to incorporate PrCa, taking into account that the energy contribution of CaPr could be comparable to that of propionic acid, with a gross energy of 3.965 Mcal/kg and an estimated metabolizable energy (ME) of 3.766 Mcal/kg (Mendoza-Martínez et al., 2017 [14]). The lambs were housed in individual roofed pens (1.5 × 1.5 m on ground floor) and dewormed with Closantel (5 mg/kg BW [body weight]) and dosed with vitamins A, D, and E (2 mg/lamb) at the beginning of this study. Feed was distributed at 08:00 and 15:00 h. Following a ten-day adaptation period (CONT), the lambs were given their assigned experimental diets for 42 days. They had unrestricted access to both feed and water, with an additional 100 g per kg of the previous day’s dry matter intake (DMI) provided to maintain a 10% feed refusal rate. The ingredients and chemical composition of dietary treatments are shown in Table 1.

Feed samples were collected daily and combined every 14 days. The diets’ dry matter (ID 934.01) and total nitrogen (ID 984.13) were analyzed using AOAC methods [19]. Neutral detergent fiber (aNDF) and acid detergent fiber (ADF) were determined following the procedure of Van Soest et al. [20], with sodium sulfite and heat-stable amylase used for aNDF analysis. The results for both aNDF and ADF included residual ash, and gross energy was measured with an adiabatic bomb calorimeter (Parr 1241 model).

### 2.3. Sample Collection and Analytical Methods

Individual rumen fluid (50 mL) was obtained using an esophageal probe on day 41 at 0700 h (previously fasted for 12 h) of the trial, and pH was measured using a pH meter (Benchtop Cole Parmer 05669–20, Vernon Hills, IL, USA). Then, ruminal fluid was filtered and immediately acidified with metaphosphoric acid (25% wt/vol). The filtered ruminal fluid was centrifuged at 12,000× *g* for 10 min. An aliquot of the supernatant was then frozen at −20 °C for later analysis of volatile fatty acids (VFAs). VFA concentrations were measured using gas chromatography (Agilent 6890, Agilent, United States, Santa Clara, CA, USA) equipped with a fused silica column (30 mm × 0.25 mm × 0.25 mm) and flame ionization detection with splitless injection. VFAs in the rumen fluid were identified by comparing retention times with known standards (Sigma-Aldrich, St. Louis, MO, United States) [21]. The concentrations of CO_2_ and CH_4_ were calculated following the method described by Russell and Strobel [22].

A total of 5 mL of ruminal fluid was placed in cryovials, flash-frozen in liquid nitrogen, and stored at −80 C until bacterial DNA was extracted. Genomic DNA was extracted using a commercial extraction kit (QIAamp Fast DNA Stool Mini Kit, Qiagen Hilden, Germany) following the manufacturer’s protocol. The DNA integrity (quality and quantity) was evaluated in agarose gel (1.5%) and a UV–Vis spectrophotometer at 260 nm (Beckman Coulter DU^®^ Spectrophotometer 730, Indianapolis, IN, USA). A segment of the 16S gene was amplified in *Fibrobacter succinogenes*, *Ruminococcus albus*, *Genus Prevotella*, *Selenomonas ruminantium*, total bacteria, and methanogenic archaea (mcrA).

The sequences of the primers used are shown in Table 2. The conditions for the PCR for each bacterium were as follows: *Genus Prevotella*: 45 s (95 °C), 45 s (58 °C), 45 s (72 °C); *Fibrobacter succinogenes*: 45 s (95 °C), 45 s (56 °C), 45 s (72 °C); *Ruminococcus albus*: 45 s (95 °C), 45 s (58 °C), 45 s (72 °C); *Selenomonas ruminantium*: 45 s (94 °C), 45 s (60 °C), 45 s (72 °C); total bacteria: 30 s (95 °C), 30 s (56 °C), 30 s (72 °C); and methanogenic archaea: 60 s (94 °C), 60 s (60 °C), 60 s (72 °C). There were 35 cycles for each bacterium.

It was verified that each primer amplified the region of interest by an endpoint PCR with a final volume of 50 µL: 19 µL distilled water, 25 µL TaqPolimerase (Thermo Scientific™, Waltham, MA, USA), 2 µL F, 2 µL R, and 2 µL ADN. The amplified fragments were visualized by electrophoresis in agarose gel (1.5%) stained with ethidium bromide and visualized under UV light. Once amplified, bands were purified using a commercial extraction kit (QIAquick Gel Extraction Kit, Qiagen^®^ Hilden, Germany) following the manufacturer’s protocol. Dilutions were prepared at four concentrations (1, 0.1, 0.01, 0.001 µg/mL) for the standard curve of each bacterium to quantify the number of genome copies per milliliter by a qTR-PCR.

The same primers were used to perform a quantitative PCR-RT using the Rotor Q5 (Qiagen) system. The final reaction contained a volume of 25 µL with 10 µL distilled water, 12 µL SYBR Green (Thermo Scientific™, Waltham, MA, USA), 1 µL F, 1 µL R, and 1 µL ADN with an unknown concentration. The amplified products were estimated using a standard curve. All qTR-PCRs were performed in duplicate. The number of copies of each bacterium was calculated using the following formula: number of copies/µL= (DNA concentration g/µL × 6.022 × 10^23^)/(size of genome (pb) × 1 × 10^9^ × 650). The number of copies was transformed to a natural log.

### 2.4. Statical Analysis

Data were analyzed using a completely randomized design with the Proc Mixed procedure in SAS 9.4 (Cary, NC, USA). The model accounted for the fixed effect of treatment and the random effect of individual lambs within each treatment. Initial body weight (BW) was included as a covariate solely for productive variables, including final BW, average daily gain (ADG), dry matter intake (DMI), and feed conversion (FC). The results were analyzed using a 2 × 2 factorial arrangement of the treatments (with CaPr and MON as the main factors). Data were analyzed using JMP software version 18 [24]. A probability of *p* < 0.05 indicated a significant difference.

## 3. Results

### 3.1. Animal Performance

Lamb performance is shown in Table 3. Dietary MON or CaPr in lamb diets did not affect (*p* ≥ 0.05) DMI, ADG, or DMI/ADG.

### 3.2. Ruminal Fermentation

There were no significant differences (*p* ≥ 0.05) in CaPr or MON doses on the ruminal pH, butyrate, propionate, acetate/propionate ratio, or CO_2_. The CaPr showed a principal effect (*p* ≤ 0.05) for decreasing acetate concentration and CH_4_ production (Table 4).

### 3.3. Ruminal Methanogenics

Table 5 shows that dietary calcium propionate increased (*p* ≤ 0.05) ruminal bacterial populations of *Fibrobacter succinogenes*, *Ruminococcus albus*, and *Selenomonas ruminantium*, whereas it reduced (*p* ≤ 0.05) methanogenic archaea. *Genus Prevotella* was affected (0.001) by MON addition to the diet. There was an interaction effect between CaPr and MON for methanogenic archaea and total bacteria.

## 4. Discussion

The results observed in the present study agree with previous studies [13,25,26,27,28,29]. A decreased feed intake was reported due to propionate infusion in the rumen, portal, or mesenteric veins [30,31]. However, Lee-Rangel et al. [13], Mendoza-Martinez et al. [27], and Cifuentes-Lopez et al. [29] mentioned that calcium propionate did not affect daily feed intake at a daily intake of 10, 20, 30, and 40 g of CaPr/kg DM in growing lambs. This effect could be observed in dairy cows, particularly in early lactation [31]. This could be explained by a negative energy balance, which induces an energy reserve mobilization (from adipose tissue); then, up to 70% of the propionic acid is transported to the liver [29,32,33]. Previously, the addition of CaPr into the diet of finishing lambs has been reported to not alter ADG [2,13,29,34], as in the present study. Likewise, dairy cows infused with propionate directly in the rumen had an increased ADG [35]. According to the authors, the increase in ADG could be related to improved energy balance and increases in plasma glucose concentrations. It is important to consider that including CaPr in diets for finishing lambs modifies the expression of hypothalamic neuropeptide Y (NPY). For NPY to be considered an appetite regulator, it must also be capable of stimulating increased DMI of ad libitum-fed sheep.

In the present study, DMI did not exhibit changes caused by MON. However, Polizel et al. [36] observed that MON decreased DMI when adding 8-to-24 mg/kg of DM monensin to female lambs, which was similar to the dosages that we used. This phenomenon was reported by Zhang et al. [37], who mentioned that the effect of ionophores could be explained by their ability to decrease rumen motility and the dilution rate of digestion of nutrients, resulting in satiety sensation and consequently a reduction in DMI, which is an important monensin effect, especially when the inclusion of concentrate is greater than 85% in diets. Some authors mentioned that monensin had been extensively used in commercial feedlot diets to increase feed efficiency and energy utilization [38,39]. Nevertheless, this was not observed in the present study, even though Susin et al. [40] mentioned that monensin promotes feed efficiency and growth rates in lambs.

Both acetate and butyrate function as precursors of long-chain fatty acids, which affects the production of H_2_, which would later be used to produce CH_4_ [41]. Propionate is ruminants’ primary substrate for gluconeogenesis and is the main hydrogen sink after CH_4_ [42]. Therefore, the acetate/propionate ratio is important because of its association with energy balance [43] and methane production from archaea.

In this study, the CaPr effect on ruminal fermentation could be attributed to the dissociation of calcium propionate [44], which provokes a dissolved ruminal propionate that affects fermentation kinetics [13], coinciding with previous studies with lambs [27] and steers [45]. In the present study, the CaPr diet contributed only to 0.003 mmol/L of propionic acid, which could not be enough to have modified the ruminal VFA contents. Lee et al. [13] observed a reduction in acetate when adding calcium propionate. Ferraro et al. [46] also observed in in vitro studies that propionate precursors increased propionate. However, an in vitro study showed that VFAs are changed by increases in propionate, butyrate, and valerate [33]; this may be explained because the in vitro conditions are in a close circuit, and the products cannot be absorbed or removed as they are in in vivo conditions.

Monensin is known to shift the acetate/propionate ratio [47], where the propionate molar percentage increases at the expense of acetate. Propionate is a gluconeogenic VFA; thus, this increases propionate availability for glucose production. Monensin also decreases the molar percentage of acetate, increases the molar percentage of propionate, and reduces the acetate/propionate ratio [46]. In this study, MON reduced methane production by 10% compared with the control, which is a similar reduction to that reported in other studies [48]. It can be explained by the effect on bacteria susceptible to ionophores, which are the leading producers of acetate, butyrate, H_2_, and CO_2_, which are substrates for methanogenesis [41]. Osorio-Teran et al. [49] reported methane reductions between 23 and 28% with slightly higher doses than those reported in the present study. In contrast, Kim et al. (26) observed a dramatic reduction in methane (53%) with doses not biologically proper for animals. Hook et al. [42] mentioned that monensin inhibits the production of CH_4_ by ruminants because inhibiting hydrogen-producing bacteria alters the ruminal VFA profile, inducing more ruminal propionate production. In this way, the reducing equivalents (H_2_) are shifted towards propionate production, and thus CH_4_ production is reduced.

Ionophores are known to capture more hydrogen and use it for propionic acid production [50], which has been associated with changes in Gram-positive bacteria; however, some molecular studies have not revealed significant shifts in relative abundance from Gram-positive bacteria in response to monensin [42]. The anti-methanogenic effects associated with the antibacterial effect have been questioned by the possible adaptation of bacteria to antibiotics [51]. In this regard, Appuhamy et al. [12] showed that the effects of reducing methane have a period of less than 30 days. In contrast, Hook et al. [52] observed that long-term supplementation with monensin (20, 90, 180 days) does not affect the rumen’s quantity or diversity of methanogens. These results suggest that the effect may be through the protozoa, as monensin is toxic [53] and protozoa hydrogenosome produces a significant amount of hydrogen; methane synthesis is directly related to the concentration of dissolved hydrogen in the rumen fluid.

It is also important to note that in several reports *Genus Prevotella* is one of the most abundant rumen bacterial genera [26], so any additive that favors their growth will be reflected in this microorganism. The addition of 10 g/kg of calcium propionate favored the presence of the genus *Fibrobacter succinogenes* and *Ruminococus albus* (cellulolytic bacteria), which can be explained by the reduction in grain, which resulted in more fibrous substrates in the diet. *Selenomonas ruminantium* was increased (*p* < 0.05) by the addition of monensin or calcium propionate (Table 5), while no effect on total bacteria was observed.

Monensin did not affect methanogenic archaea, whereas calcium propionate reduced its log copy numbers. These results are consistent with those reported by Hook et al. [52], who found no effect on methanogenic archaea by supplementing lactating cows with 24 mg/kg of monensin. Melchoir et al. [51] found no effect on heifers fed monensin on methane production. The methanogenic archaea were reduced by the CaPr effect, and Miranda et al. [14] did not detect its effect using the same dose in in vitro conditions. The mechanisms of CaPr are unclear, but it can be speculated that the effects could be related to the dissociation of calcium, changes in ruminal pH, and modifications in ruminal osmotic pressure; CaPr can increase osmotic pressure and affect bacterial growth [14], and rumen protozoa [53] and the methanogenic archaea are sensitive to changes in osmotic pressure. Besides bacteria, rumen protozoa are also affected by ruminal osmotic pressure.

## 5. Conclusions

The inclusion of monensin and calcium propionate at 10 g/kg DM did not affect performance productivity, but the addition of calcium propionate at 10 g/kg DM in finishing lamb diets changed rumen fermentative patterns and the rumen microbiome (increasing *Fibrobacter succinogenes, Ruminococcus albus,* and *Selenomonas ruminantium* and decreasing methanogenic archaea). Finally, the results indicated that a dietary combination of monensin and calcium propionate is an option to reduce methane emissions in lamb production.

## Figures and Tables

**Table 1:** Ingredient and chemical composition of dietary treatments.

	Control	MON	CaPr	MON + CaPr
Ingredient, g/kg DM				
Ground corn	400	400	300	300
Soybean meal	150	150	165	165
Molasses	90	90	120	120
Corn stover	340	340	385	385
Urea	10	10	10	10
Minerals ^a^	10	0	10	0
Minerals with monensin	0	10	0	10
Calcium propionate ^b^	0	0	10	10
Chemical composition (g/kg DM)				
Dry matter	905	896	886	891
Crude protein	122	130	111	125
Neutral detergent fiber	418	389	441	413
Acid detergent fiber	205	214	217	209
ME, Mcal/kg ^c^	2.55	2.55	2.47	2.47

^a^ Minerals: buffer 6000 g, sodium chloride 3000 g, Co 75 mg, Cu 5000 mg, Cr 200 ppb, P 4%, Fe 30,000 mg, Mn 2000 mg, Se 100 mg, I 125 mg, Zn 10,500 mg, Vitamin A 6,800,000 IU, Vitamin D 630,000 IU, Vitamin E 16,500 UI. ^b^ Ca-Pr: propionate 65%, calcium 35%, ash 35.66%. ^c^ Metabolizable energy calculated based on NRC 2007.

**Table 2:** Primers for the real-time PCRs and PCR conditions.

Target	Primer Sequence (5′-3′)	Temp. Annealing (°C)	Product Size (bp)
*Genus Prevotella* ^a^	F:GGTGTCGGCTTAAGTGCCAT R:CGGACGTAAGGGCCGTGC	58	83
*Fibrobacter succinogenes* ^a^	F:GTTCGGAATTACTGGGCGTAAA R:CGCCTGCCCCTGAACTATC	56	118
*Ruminococcus albus* ^c^	F:CCACATTGGGACTGAGACAC R:CATTATCGTCCTTTAAGACAGGAG	58	146
*Selenomonas ruminantium* ^c^	F:TAAAAGTGCGGGGCTCAAC R:TCAGCGTCAGTTACAGTCCAGA	60	123
*Methanogenic bacteria* ^c^	F:TGTCAGGTGGTGTCGGATTC R:TTGTTCAGTGCGTAGTCGTATCC	60	122
*Total bacteria* ^b^	F:GTGSTGCAYGGYTGTCGTCA R:ACGTCRTCCMCACCTTCCTC	56	110

^a^ Stevenson and Weimer [11], ^b^ Vasta et al. [23], ^c^ oligonucleotides designed by Martinez (unpublished, Laboratorio de Microbiológica Agropecuaria, UAM-Xochimilco).

**Table 3:** Effects of monensin and calcium propionate on lamb performance.

Item	Treatment				SEM	*p*-Value		
	Control	MON	CaPr	MON + CaPr		MON	CaPr	MON*CaPr
Initial Body Weight, kg	24.04	25.37	23.23	23.85	0.92	0.56	0.50	0.78
Final Body Weight, kg	31.6	32.04	30.07	31.57	1.83	0.09	0.96	0.12
Dry Matter Intake, g/day	1047	1097	1038	1070	0.02	0.14	0.51	0.75
Average Daily Gain, g/day	180	159	163	184	0.015	0.96	0.80	0.18
Feed Conversion Ratio, ADG/DMI	5.81	6.89	6.36	5.81	0.35	0.93	0.26	0.66

CaPr, calcium propionate; MON, monensin; SEM, standard error of the mean; MON*CaPr, interaction Monensin × Calcium Propionate treatments.

**Table 4:** Effects of monensin and calcium propionate on ruminal fermentation.

Item VFA (mol/100 mol)	Treatment					*p*-Value		
	Control	MON	CaPr	MON + CaPr	SEM	MON	CaPr	MON*CaPr
Acetate	65.77	65.50	65.09	63.22	0.69	0.12	0.03	0.25
Propionate	22.69	23.01	23.18	24.85	0.88	0.26	0.19	0.44
Butyrate	11.51	11.47	11.70	11.90	0.43	0.85	0.48	0.78
Acetate/propionate	2.04	2.11	2.17	2.05	0.14	0.48	0.81	0.84
Total VFA, mmol	55.07	68.38	59.05	64.18	3.18	0.36	0.98	0.47
Ruminal pH	6.69	6.46	6.74	6.67	0.09	0.10	0.16	0.38
CO_2_ (%)	55.83	55.71	55.89	55.68	0.60	0.78	0.98	0.93
CH_4_ (%)	35.69	35.52	35.04	33.49	0.56	0.13	0.02	0.22

CaPr, calcium propionate; MON, monensin; VFA, volatile fatty acid; CO_2_, carbon dioxide; CH_4_, methane; SEM, standard error of the mean; MON*CaPr, interaction Monensin × Calcium Propionate treatments.

**Table 5:** Effects of monensin and calcium propionate on ruminal bacterial populations.

	**Treatment**				**SEM**	***p*-Value**		
	**Control**	**MON**	**Ca-Pr**	**MON + PrCa**	**SEM**	**MON**	**PrCa**	MON*CaPr
*Fibrobacter succinogenes*, CYC	2.06	2.05	2.38	2.28	0.13	0.69	0.04	0.75
*Genus Prevotella*, CYC	4.27	4.69	4.40	4.78	0.13	0.001	0.43	0.88
*Ruminococcus albus*, CYC	2.64	2.84	3.29	3.29	0.12	0.44	0.001	0.41
*Selenomonas ruminantium*, CYC	2.12	2.11	2.43	2.34	0.13	0.69	0.04	0.75
*Methanogenic archaea*, CYC	2.48	2.39	2.06	2.25	0.04	0.27	0.001	<0.001
Total bacteria, CYC	7.00	7.64	7.46	7.22	0.17	0.24	0.89	0.01

CaPr, propionate of calcium; MON, monensin; SEM: standard error of the mean. Means followed by different lower-case letters in the same row are significantly different (*p* < 0.05); Data were normalized by cyclophilin B mRNA quantification; MON*CaPr, interaction Monensin × Calcium Propionate treatments.

## Data Availability

The authors will make the raw data supporting this article’s conclusions available without undue reservation.
